# Sarcopenia and self-reported markers of physical frailty in patients with osteoporosis

**DOI:** 10.1007/s11657-024-01437-9

**Published:** 2024-08-17

**Authors:** B. R. Nielsen, H. E. Andersen, P. Hovind, N. R. Jørgensen, P. Schwarz, S. H. Kristensen, C. Suetta

**Affiliations:** 1 Department of Geriatric Medicine, Amager and Hvidovre Hospital, Copenhagen, Denmark; 2 Department of Clinical Physiology, Nuclear Medicine & PET, Rigshospitalet, Glostrup, Copenhagen, Denmark; 3 Department of Clinical Physiology and Nuclear Medicine, Bispebjerg and Frederiksberg Hospital, Copenhagen, Denmark; 4 Department of Clinical Biochemistry, Rigshospitalet, Copenhagen, Denmark; 5 Faculty of Medical and Health Sciences, Copenhagen University, Copenhagen, Denmark; 6 Translational Research Centre, Rigshospitalet, Copenhagen, Denmark; 7 Department of Endocrinology, Rigshospitalet, Copenhagen, Denmark; 8 Geriatric Research Unit, Department of Geriatric Medicine, Bispebjerg and Frederiksberg Hospital, Copenhagen, Denmark

**Keywords:** Osteosarcopenia, Muscle impairment, Physical activity, Physical frailty

## Abstract

***Summary*:**

Bone and muscle impairment, named osteoporosis and sarcopenia, may co-occur with age, and patients with both disorders might exhibit physical frailty. One-hundred sixty-three patients were included. 14.2% had both disorders and presented more frequent with previous fall, reduced daily activity level, walk/balance challenges, and need of walking aid, indicating overall frailty.

**Purpose:**

In older adults, sarcopenia (muscle impairment) and physical frailty may accompany osteoporosis (bone brittleness), yet osteoporosis is typically assessed without evaluating these conditions, even though coexistence may contribute to exacerbated negative health outcomes. We aimed at evaluating the prevalence of sarcopenia and impaired muscle domains in osteoporotic patients and explore the risk of osteosarcopenia from markers of physical frailty.

**Methods:**

In Copenhagen, Denmark, osteoporotic patients aged 65 + were assessed cross-sectionally in 2018–2019. Evaluations included muscle mass, strength, and function; bone mineral density; and self-reported physical activity, fall, balance challenges, dizziness, and the need of walking aid. Low bone mass, low-energy fracture, or treatment with anti-osteoporotic medication defined patient with osteoporosis, and sarcopenia was defined by low muscle strength and mass. Osteosarcopenia was defined from the coexistence of both conditions.

**Results:**

One-hundred sixty-three patients with osteoporosis were included. Of those, 23 (14.2%) exhibited sarcopenia, hence osteosarcopenia. Hand-grip-strength, 30-s-chair-stand-test, relative-appendicular-lean-muscle-mass, and gait-speed were below cut-off levels in 21.0%, 30.9%, 28.8%, and 23.6% of the patients, respectively.

Previous fall, activity level, walk and balance challenges, and need of walking aid were statistically (or borderline) significantly more often affected in the osteosarcopenic group compared with the solely osteoporotic. Logistic regression analysis, however, revealed that only the need for walking aid significantly increased the risk of an osteosarcopenia diagnosis (odds ratio 5.54, 95% CI (1.95–15.76), *p* < 0.01).

**Conclusions:**

Sarcopenia and impaired muscle domains were frequent in osteoporotic patients, as were markers of physical frailty, indicating the need of thorough examination of osteoporotic patients.

## Introduction

Aging has a detrimental impact on the musculoskeletal system, potentially resulting in osteoporosis and sarcopenia, thus giving rise to the combined geriatric syndrome known as osteosarcopenia. The etiology of bone and muscle impairment is inherently multifaceted. Nevertheless, prevailing theories suggest a convergence of contributory factors, encompassing endocrinological and humoral influences, alongside the imperative necessity for both tissues to undergo physical stimulation [1]. Additionally, bone and muscle mass develop in parallel as they both build up during growth and decline during aging [2–4].

Global life expectancy is forecasted to increase, which is why the prevalence of osteosarcopenia, an age-related condition, is anticipated to rise as well [5–7], leading to citizen- and community-related challenges. However, probably due to differing definitions of sarcopenia [8], the reported prevalence of osteosarcopenia varies considerably among elderly home dwellings (1.5–32.2% [9–13]) as well as in hospital settings (geriatric ward: 14.2%; osteoporosis outpatient clinics: 20–65%; wards treating osteoporosis-related major fracture: 46% [14–18]).

In 2018, the European Working Group on Sarcopenia in Older People (EWGSOP2) introduced an updated guideline for diagnosing sarcopenia. This includes the application of the SARC-F questionnaire and/or clinical suspicion of sarcopenia as primary screening tools, followed by assessments of muscle strength (e.g., hand grip strength (HGS) or 30-s-chair-stand-test (CST)), and secondly muscle quantity (e.g., muscle mass measured by DXA). Lastly, the severity of sarcopenia is suggested to be evaluated by tests of physical function (e.g., gait speed (GS)) [19].

Osteoporosis is characterized by changes in the microarchitecture of the bone causing a brittle state where a fall or in severe cases even mild stress, such as bending over or coughing, may cause osteoporotic fractures [3]. The definition of osteoporosis is based on a *T*-score ≤  − 2.5 derived from a measure of bone mineral density (BMD) or the occurrence of a low-energy fracture of the spine or hip [20].

Both sarcopenia and osteoporosis are independently associated with an increased risk of falls, low-energy fractures, loss of mobility, and mortality [21–27]. Patients with both conditions are expected to have a higher risk of these outcomes [4, 28–30], although conflicting results exist [16, 26, 31, 32].

### Purpose

Given the divergent reported prevalence of osteosarcopenia, our aims were to investigate the prevalence of sarcopenia, from the EWGSOP2 guideline, and impaired single muscle domains in elderly geriatric patients suffering from osteoporosis. Furthermore, we sought to explore the phenotype of osteosarcopenic patients with the hypothesis that this group more often presents with markers of physical frailty with regards to previous falls and fractures, the need for walking aids, walking and balance challenges, dizziness, and reduced daily physical activity compared with the osteoporotic group alone. Concurrently, we will assess the risk of having sarcopenia, i.e., osteosarcopenia, based on these parameters.

These objectives all contribute to exploring whether patients treated at an osteoporosis outpatient clinic should be examined and potentially treated for sarcopenia and the subsequent markers of physical frailty alongside their established osteoporosis treatment.

## Methods

### Study design, population, and approvals

In a cross-sectional study carried out from May 2018 until November 2019, participants were recruited from a geriatric outpatient clinic specializing in osteoporosis treatment (Department of Internal Medicine M, Geriatric Section, located at Glostrup, Amager, and Hvidovre Hospital, Copenhagen, Denmark). Patients were eligible for inclusion if their age was equal to or above 65 years and if they were diagnosed with osteoporosis. There were no specific exclusion criteria. A single physician conducted the interviews, performed functional tests, and reviewed the medical report for fracture and fall, while DXA scanning was conducted at the Department of Clinical Physiology and Nuclear Medicine, Rigshospitalet, Glostrup, Copenhagen, Denmark. The Danish Ethic Committee (H-18002396) and The Danish Data Protection Agency approved the study.

### Interview

Data on self-reported markers of physical frailty from history of falls, daily challenges with walking and balance, reliance on walking aids, episodes of dizziness, previous low-energy fractures, and specific fracture types (such as spine, hip, proximal overarm, and wrist) were obtained. Validated questionnaires on future fracture risk (FRAX), daily activity (PASE), and risk of sarcopenia (SARC-F) were also administered.

### Anthropometry, bone mineral density, and body composition

Height and weight were measured, and body composition and BMD of the lumbar spine (average of L1-L4) and hip (total hip and femoral neck) were assessed using an iDXA fan-beam densitometer (GE Lunar, Madison, WI, US). *T*-scores, based on the NHANES reference, were automatically calculated from the BMD measurement. The same physician evaluated each DXA-scanning, in accordance with the 2015 ISCD guideline, where vertebral or hip DXA measurements influenced by artifacts were excluded [33].

Appendicular muscle mass was determined from whole-body DXA scanning, and the relative appendicular skeletal muscle mass was calculated by summing lean soft tissue masses for legs and arms in kg relative to height squared in meters (RALM in kg/m2).

### Muscle strength and physical function

The following examinations were conducted according to our national physiotherapy guidelines:

- HGS of the dominant hand was measured in three successive repetitions using a Jamar dynamometer (DHD-1 Digital Hand Dynamometer, Saehan 2012).
- The ability to transition from sitting to standing without using armrests was assessed by counting the repetitions in the CST.
- GS(m/sec) was measured three times over a 10 m distance at a habitual walking pace.

The highest HGS, the number of CST repetitions, and the mean GS value were included in the analyses.

### Definitions of osteoporosis, sarcopenia, and osteosarcopenia

Participants were diagnosed with osteoporosis according to WHO guidelines if their DXA scanning revealed a *T*-score ≤  − 2.5 (NHANES) or if they had experienced a low-energy fracture of the spine or hip [20]. However, in this study, though, we classified the patients suffering from osteoporosis when these criteria were met or the patients currently or previously received anti-osteoporosis treatment, in alignment with the previous categorization [2, 34].

Sarcopenia was classified into four levels according to the EWGSOP2 guideline: “No sarcopenia” from normal HGS/CST; “Probably sarcopenia” from low HGS/CST; “Confirmed sarcopenia” from low HGS/CST and additional low RALM; and “Severe sarcopenia” from low HGS/CST, low RALM, and slow GS [19]. A 2-level classification was derived by merging levels 1 and 2 as well as 3 and 4, resulting in the absence or presence of sarcopenia.

Listed below are the cut-off values recommended and applied in the data analyses:

- HGS: women < 16 kg/men < 27 kg (derived from a British healthy cohort based on the − 2.5 SD) [19, 35]
- CST: (derived from a Danish healthy cohort based on − 2 SD) [36, 37]
  - Age 60–69 y: women < 8 repetitions/men < 8 repetitions
  - Age 70–79 y: women < 7 repetitions/men > 7 repetitions
  - Age ≥ 80 y: women < 6 repetitions/men < 5 repetitions
- RALM: women < 5.5 kg/m^2^/men < 7.0 kg/m.^2^ (derived from an Australian healthy cohort based on − 2 SD) [19, 38]
- GS: both gender ≤ 0.8 m/sec [19]

Patients were categorized as having osteosarcopenia if they had both osteoporosis and sarcopenia.

### Data collection and statistics

Data were collected and stored in Research Electronic Data Capture (REDCap, version 10.3.3, 2020, Vanderbilt University, Nashville, Tennessee, USA) and exported to SAS Studio (SAS Institute Inc., Cary, NC, USA) for statistical processing.

We omitted fall data for a single outlier because of an unusually high number of falls.

Using the “univariate, means and freq procedures” in SAS, baseline characteristics and frequency of muscle impairment, previous fall and fracture and fracture types were generated and presented as means or medians according to the distribution as well as numbers, when appropriate.

Phenotypical differences between osteosarcopenic patients and patients with osteoporosis in isolation were explored using the SAS procedures “ttest, npar1way (Wilcoxon) and freq (odds ratios and chi-square measures),” when appropriate. Further, to explore the risk of having osteosarcopenia from the markers of physical frailty, we conducted logistic regressions controlled for confounders, when appropriate. The variables age, BMI, and biological sex were explored together with the markers of physical frailty of interest in this study. Variables considered mediators were not included in the models. *p* values < 0.05 were considered statistically significant.

## Results

### Population characteristics

We examined 163 patients from our osteoporosis clinic within the Department for Geriatric Medicine. The mean age of the study population was 80.1 years, predominantly comprising women (88.3%). Population characteristics are outlined in Tables 1 and 2, the latter detailing markers of physical frailty and recall data on previous fall and fracture. Approximately 43.2% had encountered a fall within the last year, while 63.2% reported daily struggles with walking and balance, of which 40.8% needed assistance from a walking aid. Additionally, subjective dizziness was noted in 35.6% of the patients. Previous fracture rate was 69.3% with vertebral compression fracture being the most frequent sub-type followed by wrist, hip, and shoulder/upper arm fracture, respectively.

**Table 1:** Population characteristics (women/men)

	Number	Mean; median*	Min	Max	SD; Q1: Q3*
Age (year)	144/19	80.2/82*	67/65	98/88	6.2/74: 83*
BMI (kg/m^2^)	144/19	24.6/23.9*	13/17.4	40.9/47.5	4.6/18.6: 28.0*
L1–L4 *T*-score	119/18	− 2.2/ − 1.8*	− 5.4/ − 5.1	1.3/1.1	1.1/ − 3.1: − 0.4*
Left neck *T*-score	122/18	− 2.2/ − 2.8*	− 4.2/ − 4.4	0.6/2.3	0.7/ − 3.5: 2.0*
Left total hip *T*-score	122/18	− 2.0/ − 2.0*	− 4.0/ − 4.4	− 0.2/3.0	0.8/ − 3.4: − 1.5*
FRAX: major osteoporotic fracture (%)	142/18	33.0*/18.0*	14.0/8.7	77.0/44.0	26.0: 42.0*/13.0: 34.0*
FRAX: hip fracture (%)	142/18	14.0*/11.5*	1.8/2.7	70.0/38.0	8.9: 24.0*/6.7: 21.0*
SARC-F (point)	144/19	3*/2*	0/0	10/10	1: 5*/1: 7*
PASE (point)	144/19	64.0*/94.2*	0/0	229.5/221.0	37.7: 114.0*/0: 129.0*
Hand grip strength (kg)	144/18	20.1/28.1	8.0/15.8	36.7/45.8	5.2/7.5
30-s chair stand test (repetitions)	144/18	10.5/9.5*	0/0	20/16	3: 13*/5:14*
Habitual gait speed (m/s)	142/18	1.0/1.0*	0.2/0.2	1.8/1.4	0.3/0.6: 1.2*
Relative appendicular lean mass (kg/m^2^)	144/19	6.1/6.4*	3.7/5.2	9.7/11.9	0.9/6.2: 7.3*

*Min* minimum, *Max* maximum, *SD* standard deviation, *Q1* lower quartile, *Q3* higher quartile, *kg* kilogram, *m* meter, *L* lumbar vertebrae, *FRAX* fracture risk assessment tool, *SARC-F* strength, assistance with walking, rising from a chair, climbing stairs, and falls, *PASE* physical activity score for the elderly, *s* seconds

**Table 2:** Fall, fracture, and frailty

	Number	Yes (%)
History of fall (last 12 months)	162	70 (43.2)
Daily walk and balance challenges	163	103 (63.2)
Need of walking equipment	163	46 (28.2)
Dizziness	163	58 (35.6)
History of low-energy fracture	163	113 (69.3)
Type of fracture		
Wrist	113	60 (53.1)
Shoulder/upper arm	113	15 (13.3)
Hip	113	22 (19.5)
Spine	113	70 (61.9)

### Prevalence of impaired muscle domain and (osteo)-sarcopenia

The muscle domains HGS, CST, RALM, and GS were found to be below cut-off levels in 21.0%, 30.9%, 28.8%, and 23.6% of the patients, respectively. Forty-one percent exhibited impairment in either or both HGS and CST (indicative of probable sarcopenia), while 14.2% presented with sarcopenia, signifying osteosarcopenia in these instances. Among these, 47.8% were classified as severe (Table 3).

**Table 3:** Muscle Impairment

	Number	Yes (%)
Low HGS	162	34 (21.0)
Low CST	162	50 (30.9)
Low HGS and/or low CST	162	67 (41.4)
Low RALM	163	47 (28.8)
Slow GS	160	38 (23.8)
4-level-sarcopenia		
No sarcopenia	160	95 (59.4)
Probably sarcopenia	160	42 (26.3)
Confirmed sarcopenia	160	12 (7.5)
Severe sarcopenia	160	11 (6.9)
2-level-sarcopenia		
No sarcopenia	162	139 (85.8)
Sarcopenia	162	23 (14.2)

*HGS* hand grip strength, *CST* chair stand test, *RALM* relative appendicular lean mass, *GS* gait speed

### Osteosarcopenia versus osteoporosis and prediction of the former

The osteosarcopenic and osteoporotic patients were comparable in age (79.8 years, 95% CI (76.5–83.1) vs. 80.1 years, 95% CI (79.1–81.1), *p* = 0.85), but those with osteosarcopenia exhibited, on average, a lower BMI of 21.4 kg/m^2^ (95% CI (19.8–22.9)) compared to 25.0 kg/m^2^ (95% CI (24.2–25.7)) with osteoporotic patients (*p* < 0.001).

Despite both groups having clinical osteoporosis, patients with concurrent sarcopenia showed lower *T*-scores for both the total hip (− 2.65, 95% CI (− 3.17 to − 2.13) vs. − 1.87, 95% CI (− 2.02 to − 1.73), *p* < 0.001) and neck (− 2.90, 95% CI (− 3.40 to − 2.32) vs. − 2.21, 95% CI (− 2.34 to − 2.09), *p* < 0.05), as well as a higher risk of future hip fractures based on FRAX (21.0%, Q1:Q3 (14.0:29.0) vs. 13.1%, Q1:Q3 (8.5:20.5), *p* < 0.01). However, there were no differences in lumbar spine *T*-score (− 2.2, 95% CI (− 2.4 to − 1.9) vs. − 2.1, 95% CI (− 2.5 to − 1.81), *p* = 0.95) or FRAX for major osteoporotic fractures (38.0%, Q1:Q3 (29.0:45.0) vs. 31.5%, Q1:Q3 (25.0:41.0), *p* = 0.09).

As depicted in Table 4, osteosarcopenic patients exhibited more often markers of physical frailty evidenced by lower activity scores, more frequent daily walking and balance challenges, and a higher reliance on walking aids, but there was no discernible difference in reported dizziness. Previous falls were more prevalent in the osteosarcopenic group (60.9% vs. 39.9%), albeit only showing borderline statistical significance (*p* = 0.06) when not Bonferroni corrected (Table 4). Previous fracture rates were comparable between the two groups (Table 4). Impairments in walking and balance, as well as the need for walking aid, were associated with approximately a 4.5-fold increase in the risk of having sarcopenia. Previous falls increased the risk by nearly 2.5-fold, though only achieving borderline significance. However, prior fracture history or dizziness did not impact the risk of sarcopenia (Table 4).

**Table 4:** Osteosarcopenia versus osteoporosis

Focus grouping	Unit	Osteosarcopenia number (%); median (Q1: Q3)*	Osteoporosis number (%); median (Q1: Q3)*	Odds ratio (95% CI); difference in median*	*P* value (Bonferroni)	Logistic regression estimate (95% CI)	*P* value (Bonferroni)
Previous fall and fracture							
Fall (within the last year) (*n* = 161)	Yes	14 (60.9)	55 (39.9)	2.35 (0.95–5.80)	0.06^1^ (0.12)	Model 1; 2.86 (1.10–7.63)	** < 0.05**; 0.07
	No	9 (39.1)	83 (60.1)				
Low-energy fracture (*n* = 162)	Yes	17 (73.9)	95 (68.4)	1.31 (0.48–3.56)	0.60^1^ (1)	Model 2; 2.01 (0.64: 6.33)	0.23 (1)
	No	6 (26.1)	44 (31.7)				
Actual frailty parameters							
PASE (*n* = 162)	Points	35 (4.2: 70.0)*	70 (45: 122.7)*	35*	** < 0.01**^2^; **(< 0.01)**	Model 3; 0.87 (0.76–0.99)	** < 0.05**; (0.13)
Daily walk and balance challenges (*n* = 162)	Yes	20 (87.0)	82 (59.0)	4.63 (1.31–16.33)	** < 0.05**^1^; **(< 0.05)**	Model 4; 4.20 (0.94–18.84)	0.06; (0.24)
	No	3 (13.0)	57 (41.0)				
Need of walking equipment (*n* = 162)	Yes	13 (56.5)	32 (23.0)	4.35 (1.74–10.84)	** < 0.001**^1^; **(< 0.01)**	Model 5; 5.54 (1.95–15.76)	** < 0.01**; **(< 0.01)**
	No	10 (43.5)	107 (77.0)				
Dizziness (*n* = 162)	Yes	8 (34.8)	50 (36.0)	0.95 (0.38–2.39)	0.91^1^; (1)	Model 6; 0.95 (0.38–2.40)	0.91; (1)
	No	15 (65.2)	89 (64.0)				

Abbreviations: *Q1* lower quartile, *Q3* higher quartile, *CI* confidence interval, *n* number, *PASE* physical activity score of the elderly

Statistical methods for differences: ^1^chi-square; ^2^non-parametric, Wilcoxon

Model 1: Mediator: fracture. Potential confounders: BMI, age, and gender. Controlled for BMI and age

Model 2: Potential confounders: BMI, age, gender, and fall. Controlled for BMI, age, and fall

Model 3: Potential confounders: BMI, gender, age, walking equipment, walk and balance challenges and dizziness. Controlled for walking equipment and walk og balance challenges. Estimate for 10 point increase in PASE

Model 4: Mediator: PASE. Potential confounders: BMI, age, gender, walking equipment and dizziness. Controlled for all confounders listed

Model 5: Mediators: PASE, daily walk and balance challenges and dizziness. Potential confounders: BMI, age, and gender. Controlled for alle confounders listed

Model 6: Mediators: BMI, walk and balance challenges, and PASE. Potential confounders: age, gender, and walking equipment. Controlling for potential confounders not necessary

In logistic regression analysis, when adjusted for confounders as appropriate, we found that previous falls and the reliance on walking aid increased the risk of osteosarcopenia by 2.9- and 5.5-fold, respectively. However, only the need for a walking aid remained statistically significant after the Bonferroni correction (multiple testing within each focus groups). Additionally, a 10-point increase in PASE reduced the risk of osteosarcopenia by 13.3%, though this reduction did not maintain significance after the Bonferroni correction.

## Discussion

While osteoporosis outpatient clinics primarily focus on diagnosing and treating osteoporosis, recognizing the interconnectedness of bone and muscle health suggests that addressing both could mitigate an emerging challenge of global significance. The present osteoporosis cohort was notably physical frail beyond just having fragile bones, as a substantial proportion exhibited impaired muscle function (~ 20–40%), daily challenges with walking and balance (~ 63%), reliance on walking aids (~ 28%), and a high incidence of previous falls (~ 43%), although a higher proportion of fallers (62.9%) has been reported in osteoporosis cohorts [39]. The notion of a weakened cohort is further supported by high average FRAX scores when compared with the NOGG 2021 guideline (MOF: high risk ~ 20%, very high risk ~ 30%; HF: high risk ~ 5%, very high risk ~ 9%) [40] and relatively low mean PASE scores as normal ranges of 60–115 points and 100–145 points for women and men, respectively, are reported [41]. Surprisingly, low median SARC-F scores were obtained indicating an overall low risk of sarcopenia as a score ≥ 4 points is reported to be predictive of sarcopenia and poor outcome [42]. We report 14.2% of our study population to present with sarcopenia—a clinically significant quantity—albeit lower compared with previous findings from osteoporotic outpatient clinics, where other has reported the coexistence of sarcopenia ranging from 20 to 65% [14, 15]. However, our findings are in line with recent data from a fall and fracture clinic in Melbourne as 25.6% had osteosarcopenia defined from the additive of probably, confirmed, and severe sarcopenia, which is similar to our findings of 26.3% [43].

Self-reported walk and balance challenges were significantly higher in the osteosarcopenic group, which is in line with a study of 306 patients aged ≥ 65 years from a fall and fracture clinic [44]. Poorer balance (from high ellipse area and sway) and worse gate parameters (from cadence, step length, and stride length) in the osteosarcopenic patients versus patients with osteoporosis only were reported. In patients with poor balance, we found that 56.6% of the osteosarcopenic patients needed walking aid. This was lower as compared with data from the SAGE study, where both the osteoporotic and the osteosarcopenic group needed walking equipment in about 70% of the cases. This cohort, however, included patients admitted to a geriatric ward, who are expected to be more vulnerable compared to outpatients [17]. Nevertheless, we found that the risk of being diagnosed with osteosarcopenia was higher when patients were dependent upon support from a walking aid. Furthermore, being more daily active reduced the likelihood of being osteosarcopenic, which is supported by Curcio et al. [45], who found a significantly lower PASE score in the sarcopenic part of 420 older adults admitted to a comprehensive geriatric assessment center.

In this study, we found that a previous fall increased the likelihood of being osteosarcopenic at the time of assessment, whereas fractures did not. As our data are retrospective and cross-sectional, we cannot predict future falls or fractures from the presence or absence of sarcopenia. However, the 10-year probability of hip fracture from FRAX was statistically higher in the osteosarcopenic group, indicating an association with the measured muscle parameters. In the MrOS study, it was explored whether adding sarcopenia measures altered the risk of future fracture beyond that given from the BMD of the femoral neck. After adjustment for age and BMD, sarcopenia, defined according to the EWGSOP2 guidelines, had a predictive value for major osteoporotic fracture (confirmed and severe sarcopenia) and hip fracture (severe sarcopenia), although slightly attenuated. The authors therefore concluded that a predictive value from the additional inclusion of physical performance measures was present [8]. With this in mind, we expected increased fracture risk in our sarcopenic patients.

Overall, markers of physical frailty in our osteosarcopenic group mirror findings from the Hertfordshire cohort study, where the likelihood of being frail from the Fried definition was higher in the osteosarcopenic patients versus both sarcopenic and osteoporotic patients alone (ORs: osteosarcopenia, 26.3; sarcopenia, 8.3; and osteoporosis, 2.6) [34]. Indeed, including standardized frailty scores as the Fried definition [46] or the CFS [47] could add clinically meaningful information.

Our study has both strengths and limitations. The application of the newest European sarcopenia definition is a strength, as our findings are more comparable with other recent studies. The wide range of previously reported osteosarcopenia prevalence internationally reflects variations in guidelines and cut-off limits for sarcopenia assessments as pointed out by Harvey et al. [8] where the prevalence of sarcopenia varied from 0.5 to 35% depending on the 11 different guidelines applied. This issue has given rise to a resent published conceptual definition of sarcopenia (GLIS), which will serve to develop a worldwide operational definition [48]. Our choice of the EWGSOP2 approach was based on its clinical relevance, feasibility in our setting, and inclusion of the recommended cut-off point [19]. However, no cut-off points were tabulated for the 30-s-CST, but local reference data were suggested when possible. We therefore applied CST cut-off limits (− 2 SD) generated from a Danish cohort aged 20–93 years [36, 37], which were in line with cut points presented by Jonas et al. [49].

The biological sex distribution in our study population consisted of 88.3% women and 11.7% men, reflecting that osteoporosis is more frequent in women. Nevertheless, the number of men was lower than expected compared to data from Denmark from 2017 where 17.6% of this cohort with osteoporosis were men [50]. In general, sex-stratified analyses were not deemed essential given that osteoporosis definition is not sex-specific, and sarcopenia definitions are controlled by different cut-off limits for women and men, but we did control for biological sex in the logistic regressions.

Due to our cross-sectional design, we were not able to predict future fall or fracture, which is needed, as pointed out by Teng et al. [51] in a recent meta-analysis, where osteosarcopenia was associated with an elevated risk of falls, fractures, and mortality compared to controls with healthy bone and muscle. However, due to insufficient data, it was not feasible to compare osteosarcopenia with sarcopenia or osteoporosis alone. This, together with our findings, underscores the need for prospective studies to ascertain the true risks of falls and fractures in osteoporotic patients versus patients with osteosarcopenia or markers of physical frailty to establish integrated treatment of patients with markers of physical frailty and sarcopenia alongside their osteoporotic condition. One potential clinical avenue could involve the development of pharmaceuticals targeting both disorders or implementing physiotherapeutic interventions such as resistance and balance training alongside anti-osteoporotic treatment to prevent the sequelae of osteosarcopenia.

## Conclusion

The co-existence of sarcopenia and osteoporosis was present in 14.2% of our study population, and these patients exhibited more often markers of physical frailty regarding previous falls, lower daily activity, increased subjective walk, and balance challenges with the need of walking aid, lower hip *T*-scores, and a higher hip FRAX scores compared to patients with osteoporosis only. Our results suggest that in an osteoporosis outpatient clinic, attention should be paid to patients presenting with markers of physical frailty, as they may have sarcopenia concurrent with osteoporosis. Considering the relatively frequent coexistence of sarcopenia and osteoporosis—osteosarcopenia—we recommend that elderly patients referred to an osteoporosis outpatient clinic should undergo assessment, evaluation, and treatment for both bone and muscle weakness as previously put forth by Kirk et al. [52]. This assessment could advantageously be supplemented by standardized scoring of physical frailty so that relevant interventions such as dual medical targeting (when or if possible, in the long term), protein supplementation, as well as resistance and balance training, could be initiated.
